# Brimonidine prevents axonal and somatic degeneration of retinal ganglion cell neurons

**DOI:** 10.1186/1750-1326-6-4

**Published:** 2011-01-13

**Authors:** Wendi S Lambert, Lupe Ruiz, Samuel D Crish, Larry A Wheeler, David J Calkins

**Affiliations:** 1 The Vanderbilt Eye Institute, Vanderbilt University Medical Center, Nashville, TN 37205, USA; 2 Allergan, Inc., Irvine, CA 92623, USA

## Abstract

**Background:**

Brimonidine is a common drug for lowering ocular pressure and may directly protect retinal ganglion cells in glaucoma. The disease involves early loss of retinal ganglion cell transport to brain targets followed by axonal and somatic degeneration. We examined whether brimonidine preserves ganglion cell axonal transport and abates degeneration in rats with elevated ocular pressure induced by laser cauterization of the episcleral veins.

**Results:**

Ocular pressure was elevated unilaterally by 90% for a period of 8 weeks post- cauterization. During this time, brimonidine (1mg/kg/day) or vehicle (phosphate-buffered saline) was delivered systemically and continuously via subcutaneous pump. Animals received bilateral intravitreal injections of fluorescent cholera toxin subunit β (CTB) two days before sacrifice to assess anterograde transport. In retinas from the vehicle group, elevated pressure induced a 44% decrease in the fraction of ganglion cells with intact uptake of CTB and a 14-42% reduction in the number of immuno-labelled ganglion cell bodies, with the worst loss occurring nasally. Elevated pressure also caused a 33% loss of ganglion cell axons in vehicle optic nerves and a 70% decrease in CTB transport to the superior colliculus. Each of these components of ganglion cell degeneration was either prevented or significantly reduced in the brimonidine treatment group.

**Conclusions:**

Continuous and systemic treatment with brimonidine by subcutaneous injection significantly improved retinal ganglion cell survival with exposure to elevated ocular pressure. This effect was most striking in the nasal region of the retina. Brimonidine treatment also preserved ganglion cell axon morphology, sampling density and total number in the optic nerve with elevated pressure. Consistent with improved outcome in the optic projection, brimonidine also significantly reduced the deficits in axonal transport to the superior colliculus associated with elevated ocular pressure. As transport deficits to and from retinal ganglion cell projection targets in the brain are relevant to the progression of glaucoma, the ability of brimonidine to preserve optic nerve axons and active transport suggests its neuroprotective effects are relevant not only at the cell body, but throughout the entire optic projection.

## Background

Glaucoma is a chronic disease that causes vision loss through the degeneration of retinal ganglion cell (RGC) neurons and their axons in the optic nerve [1,2]. While age is an important risk factor, the only modifiable risk factor and sole target for clinical intervention is elevated intraocular pressure (IOP) or ocular hypertension (OHT) [3]. Lowering IOP generally slows progression in glaucoma [3], but does not necessarily stop degeneration [4]. Thus, there is great interest in identifying neuroprotective agents as potential therapies [5,6].

Brimonidine (BMD; UK14304, Alphagan) is a non-selective α2-adrenergic receptor agonist currently used as a treatment to lower IOP in glaucoma. An effect of BMD is to decrease aqueous humor production by inhibition of adenylate cyclase inhibition, which lowers cAMP levels [7]. The drug also increases uveoscleral outflow due to prostaglandin release and/or ciliary muscle relaxation [8]. Independent of its IOP-lowering properties, BMD is neuroprotective for RGCs in various injury models [9-13]. Potential mechanisms underlying these effects include the inhibition of glutamate release, regulation of calcium influx in the inner retina, modulation of NMDA receptor signalling in RGCs, and upregulation of trophic factor expression [14-16]. Because of its dual action to lower IOP and perhaps protect against neuronal injury, BMD may hold promise in the treatment of glaucoma and other optic neuropathies [17].

Following acute ischemic injury, BMD preserves axonal transport along RGC axons from the retina to the superior colliculus [18]. This is intriguing in the context of glaucoma, as recent evidence indicates axon-specific mechanisms play an early role in the pathology of the disease [19-21]. Here we investigated whether systemic treatment with BMD by continuous subcutaneous injection improves axon survival in the optic nerve and anterograde transport to the colliculus with acute OHT.

## Results

### Systemic brimonidine treatment does not affect IOP

**Figure 1:**
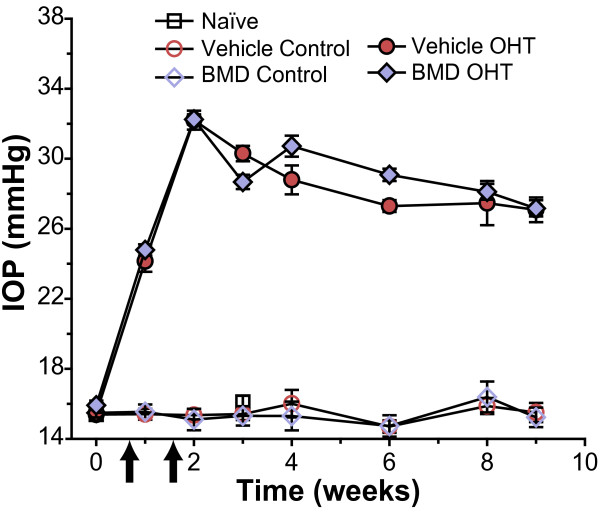
**Systemic BMD does not affect IOP**. Ocular pressure in mmHg as measured using TonoLab following laser photocoagulation of episcleral veins at two time points (arrows). Systemic BMD (1 mg/kg/day) delivered via subcutaneous osmotic pump did not affect IOP for control or OHT eyes compared to vehicle treatment (p ≥ 0.6; mean ± SEM; n = 16 eyes per group).

We induced OHT unilaterally in rats using laser photocoagulation of episcleral and limbal veins as described below. Systemic treatment with either BMD (1 mg/kg/day) or vehicle (phosphate-buffered saline, PBS) began at the time of initial IOP elevation and continued for the 8 week period of elevation. We chose this dosing regimen based on previous experiments examining the effects of systemic BMD on RGC survival with elevated ocular pressure [11]. Eyes without laser injury (naïve, vehicle control, BMD control) had an average IOP of 15.5 ± 0.1 mmHg that did not differ between groups (p = 0.59; Figure 1). One week following induction of OHT, IOP reached a peak of 32.3 ± 0.1 mmHg in both vehicle and BMD OHT eyes (p = 0.96). Thereafter IOP remained elevated by 90 ± 6% in the vehicle group and by 91 ± 7% in the BMD group compared to the control eye (p = 0.85); neither changed significantly over the experimental period (p ≥ 0.2). Thus, systemic delivery of BMD by subcutaneous osmotic pump had no effect on IOP (Figure 1).

### Brimonidine prevents loss of RGCs with OHT

**Figure 2:**
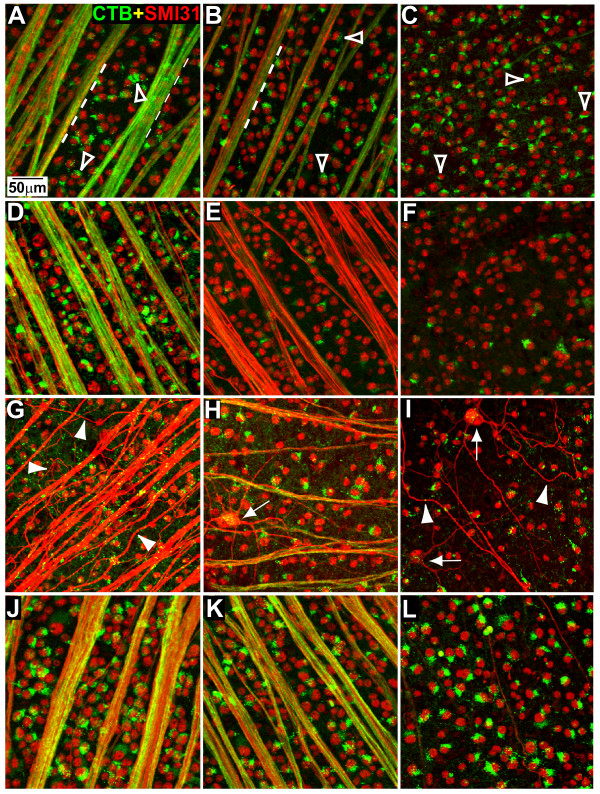
**BMD preserves RGC morphology in retinas exposed to OHT**. (A-C) Representative confocal images of whole-mounted naïve rat retina demonstrating co-localization of CTB (green) with phosphorylated heavy-chain neurofilament (SMI31; red) in RGC axons (dashed lines) and somas (open arrowheads). Similar co-localization was observed in vehicle (D) and BMD (J) control retinas. Vehicle OHT retinas (E-I) demonstrated fewer CTB + RGC somas and axons, dystrophic axons (arrowheads), and swollen RGCs with dendrites accumulating SMI31 (arrows). In BMD OHT retinas (K, L), co-localization of CTB and SMI31 was similar to that in naïve and control retinas. Distance from optic disc is 1 mm (A, D, E, G, J, K), 2 mm (B, H, I), or 4 mm (C, F, L).

We compared the localization of CTB and phosphorylated heavy-chain neurofilaments (SMI31) in whole-mounted retinas from naïve, vehicle and BMD rats (Figure 2). In naïve, vehicle and BMD control retinas, nearly every RGC labelled by SMI31 also demonstrated intact CTB uptake (Figure 2A-D,G). Vehicle OHT retinas contained far fewer CTB+ and SMI31 + RGCs (Figure 2E,F). In these retinas, RGC axons also contained less CTB, indicative of diminished active uptake and transport, and more intense concentration of SMI31, suggesting accumulation. Vehicle OHT retinas also showed obvious axonal dystrophy, swelling of RGC somas, and accumulation of SMI31 in RGC dendrites. In contrast, OHT retinas from BMD treated rats appeared similar to naïve and control retinas with respect to SMI31 and CTB localization (Figure 2H,I). Few, if any, dystrophic axons or swollen RGCs were observed in these retinas.

**Figure 3:**
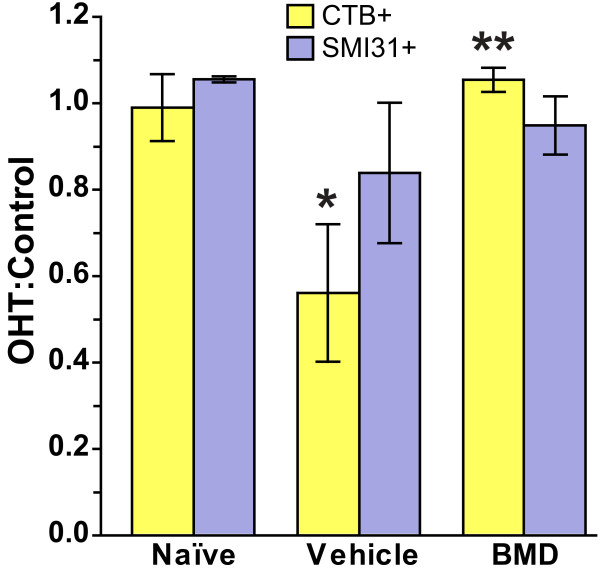
**BMD preserves CTB uptake by RGCs during OHT**. Bar chart shows CTB+ and SMI31+ RGCs as the ratio of OHT to control retina for the vehicle- and BMD-treated rats (mean ± SEM; n = 6 retina each). The naïve group is represented simply as the ratio of the right to left retina. In the vehicle group, OHT decreased the number of CTB+ RGCs so that the ratio was significantly less than one (*, p = 0.01). Treatment with BMD improved CTB uptake in OHT retinas compared to vehicle (**, p < 0.001). The number of SMI31+ RGCs in vehicle-treated retinas was also decreased by OHT, but this result was not significant. The ratio of OHT to control eye for both CTB+ and SMI31+ RGCs was identical for the naïve and BMD-treated rats (p ≥ 0.11).

Next we quantified the number of SMI31+ and CTB+ RGCs in each group. For simplicity, we represent the RGC counts as the ratio of the right to left retina for the naïve group and as the ratio of OHT to control retina for the vehicle and BMD groups (Figure 3). As expected, for the naïve group the ratios for both SMI31 and CTB counts were identical to one, indicating no difference between the right and left eyes (p ≥ 0.22). In the vehicle group, the number of CTB + RGCs in the OHT retina was only 56% that of the control retina, which was a significant reduction (p = 0.01). BMD completely prevented this decrease: the ratio of the OHT to control eye was the same as the naïve group (p = 0.42). Compared to the vehicle group, this was a dramatic improvement of 200% (p < 0.001). In terms of SMI31+ RGCs, in the vehicle group, the number in the OHT retina was 84% that of the control retina, which was not significant (p = 0.26). In the BMD group, the number of SMI31+ RGCs was the same for both eyes and not distinguishable from naïve (p = 0.11).

**Figure 4:**
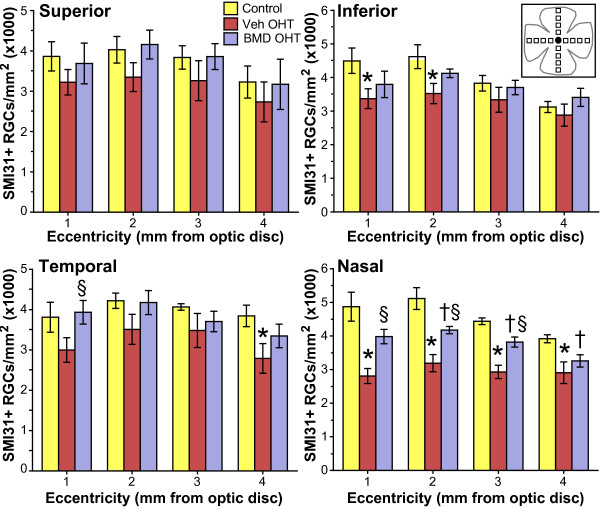
**BMD prevents modest RGC somatic loss with OHT**. RGC density is shown for control, vehicle- and BMD-treated groups across eccentricity (mean ± SEM; n = 6 retina each). RGC density decreased with OHT in vehicle retinas compared to control at all locations in each quadrant, with significant decreases indicated (*). In BMD-treated rats, RGC density with OHT was the same as control retinas except at 3 locations in the nasal quadrant (†, p ≤ 0.02) and was significantly higher than vehicle OHT retinas at locations in both temporal and nasal quadrants (§, p ≤ 0.05). Inset: Diagram showing locations in retinal quadrants 1-4 mm eccentric from the optic disc (black circle) for which SMI31+ RGCs were quantified. Size of each field is 0.101mm^2^.

Figure 3 indicates a large variability for the vehicle group in terms of the effects of OHT on RGC number, with standard errors 25 - 30% of the mean for both CTB+ and SMI31+ cells. To determine if the variability was retinotopic, as in other models [22-25], we quantified SMI31+ RGCs along the midline of each retinal quadrant as a function of eccentricity from the optic disc (Figure 4). In naïve retinas RGC density ranged from 2,900 to 5,400 RGCs/mm^2 ^with a mean across quadrants of 4000 ± 170 RGCs/mm^2^. This is comparable to published values for RGC density for this species [25,26]. The number of labelled RGCs in vehicle control retinas was slightly higher for the inferior and nasal quadrants (4600 ± 440 RGCs/mm^2^), but this difference was not significant compared to naïve (p ≥ 0.1). Across quadrants, we found no difference in RGC density between vehicle control (4200 ± 330 RGCs/mm^2^) and BMD control (4025 ± 174 RGCs/mm^2^) retinas (p ≥ 0.2), so these were combined in the comparisons that follow (Figure 4).

For all retinal quadrants, the density of SMI31+ RGCs in the control retinas peaked within 1-2 mm of the optic nerve head (3,400 to 5,400 RGCs/mm^2^) and decreased slightly (17 to 18%) at 4 mm out (Figure 4). In the superior retina of the vehicle group, OHT decreased RGC density by 15 to 17% across eccentricity compared to control eyes. RGC density in the same sector for the BMD OHT retinas was 14 - 24% higher than the vehicle OHT retinas; neither group was significant different than the corresponding control eye (p ≥ 0.2). In the inferior quadrant of vehicle retinas, OHT decreased RGC density 14 - 25% compared to control retinas with significant reductions of 25% and 23% observed at 1 and 2mm eccentric, respectively (p ≤ 0.05). For the BMD group in this quadrant, RGC density did not change with OHT compared to control (p ≥ 0.25). In the temporal quadrant for vehicle retinas, OHT decreased RGC density 14 - 27% compared to control; at 4mm eccentricity this decrease (27%) was significant (p = 0.04). For the BMD group in this quadrant, RGC density in OHT retinas was similar to control retinas at all eccentricities (p ≥ 0.2). The 31% improvement in RGC density compared to vehicle OHT retinas was significant at 1 mm from the optic disc (p = 0.05). Finally, RGC loss in vehicle OHT eyes was most dramatic in the nasal retina, where RGC density decreased at every location. Decreased density compared to control retinas ranged from 42% nearest the optic disc to 26% at 4 mm eccentricity (p ≤ 0.02). BMD treatment afforded partial rescue of RGCs in this quadrant, with significant increases of 30-42% at 1-3 mm eccentricity compared to vehicle OHT retinas (p ≤ 0.005). However, only at 1 mm was RGC density in the BMD OHT group comparable to the control group (p = 0.09).

### Brimonidine protects RGC axons

**Figure 5:**
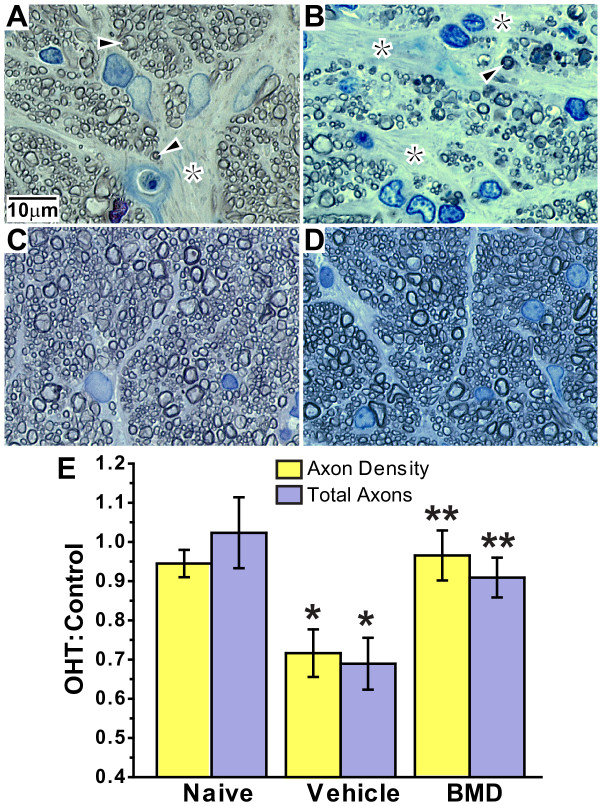
**BMD preserves RGC axon morphology, density and total number following OHT**. High-magnification light photomicrographs demonstrating degenerating axon profiles (arrowheads) and gliotic scars (asterisks) in cross-sections of vehicle OHT optic nerves (A and B). Like vehicle control nerve (C), nerves from BMD OHT rats (D) had significantly fewer degenerating profiles. (E) Axon density and total axon number expressed as the ratio of OHT to control nerve for vehicle and BMD groups; naïve group is the ratio of the right to left nerve (mean ± SD; n = 16 each). Density and axon number decreased in vehicle treated rats so that the ratio for each differed significantly from one (*, p ≤ 0.02). BMD treatment improved both outcome measures compared to vehicle (**, p = 0.011).

We compared RGC axon morphology and survival after OHT in cross sections of vehicle- and BMD-treated optic nerves. Vehicle OHT nerves (Figure 5A and 5B) displayed degenerating axon profiles and gliotic scarring compared to vehicle control nerves (Figure 5C). In contrast, BMD OHT nerves were similar in appearance to control nerves, with few if any degenerating profiles (Figure 5D). We quantified axon density (axons/mm^2^) and total number of axons for each group. Once again for simplicity, these are shown as the ratio of the right to left nerve for the naïve group and as the ratio of the OHT to control nerve for the vehicle and BMD treated groups (Figure 5E). As expected, both ratios were identical to one for the naïve, indicating no difference between the two eyes. For the vehicle group, axon density was 25% less and the number of axons 33% less in the OHT nerve compared to control, yielding ratio that differed significantly from one (p ≤ 0.03). Conversely, for BMD-treated animals both axon density and axon number were similar between the OHT and control nerves, yielding ratios identical to those for naïve rats (p ≥ 0.3). Both measures were considerably improved compared to vehicle-treated rats (p = 0.011).

### Brimonidine substantially restores anterograde transport

**Figure 6:**
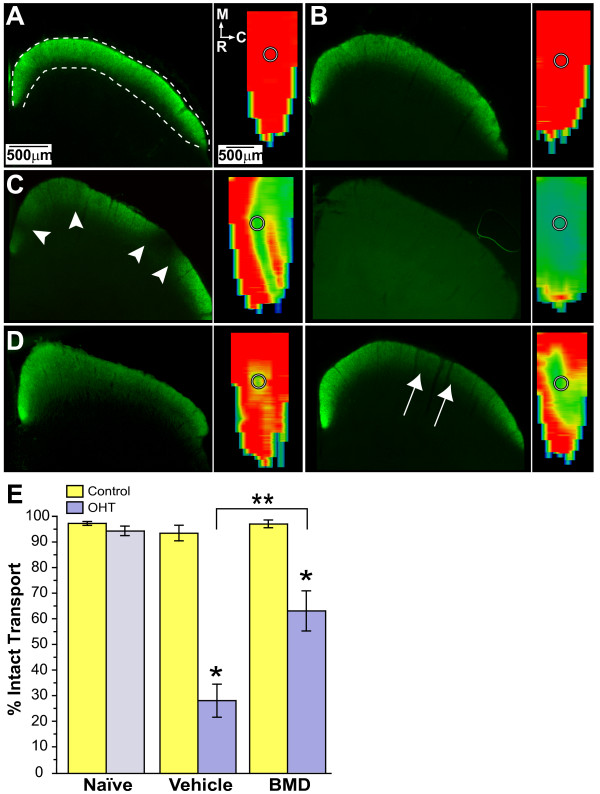
**BMD partially preserves anterograde transport following acute IOP elevation**. (A) Representative cross-section through medial superficial superior colliculus (left, outlined) from naïve rat showing normal RGC anterograde transport of CTB (green). Corresponding retinotopic map reconstructed from serial cross-sections through the colliculus (right) shows representation of optic disc gap (circle). Colorimetric scale indicates levels of transport from 100% (red) to 50% (green) to 0% (blue). (B) Comparable colliculus section (left) and retinotopic map (right) corresponding to vehicle control eye also shows intact transport. (C) Sections of colliculus corresponding to OHT eye in vehicle group show deficits ranging from sectorial (arrowheads on left) to complete loss (right). Corresponding retinotopic maps were 58% (left) and 7% (right) intact, respectively. (D) Colliculus from OHT eye in BMD-treated rats show range of rescued transport from complete (left) to modest (85% intact, right). (E) CTB transport in colliculus calculated as the fraction of the retinotopic map with ≥70% maximum signal for three groups (mean ± SEM; n = 11 each). Naïve group represents transport to the left and right colliculi. OHT decreased CTB transport 70% in vehicle-treated rats (*, p = 0.001) but only 35% in BMD-treated animals (*, p = 0.004). BMD significantly improved transport with OHT when compared to vehicle eyes (**, p = 0.002).

We compared anterograde transport of CTB from the retina to the superior colliculus in naïve, vehicle-, and BMD-treated animals (Figure 6). Complete CTB labelling of the RGC-recipient zone was observed in colliculi from naïve eyes (Figure 6A) and in colliculi from control eyes in the vehicle group (Figure 6B). The corresponding retinotopic maps for these colliculi were reconstructed from serial sections. These showed complete representations of CTB signal except for the retinal optic disc gap, which contains no RGCs. Complete labelling of colliculi from control eyes of the BMD-treated group was also observed (data not shown). In contrast, colliculi from vehicle OHT eyes demonstrated severe deficits in CTB signal, ranging from a retinotopic hemifield (Figure 6C, left) to nearly complete loss (Figure 6C, right). OHT in the BMD group had far less affect on CTB transport to the colliculus, yielding retinotopic representations ranging from complete (Figure 6D, left) to mild deficits (Figure 6D, right). When quantified as the fraction of intact retinotopic map (Figure 6E), as expected CTB transport to the colliculus was similar for the two eyes of the naïve group (p ≥ 0.18). Similarly, there was no difference in intact transport between naïve colliculi and colliculi from the control eyes for both the vehicle and BMD groups (p ≥ 0.49). With OHT for vehicle rats, the average deficit in CTB transport was 71.8 ± 6.4%. With a range of intact transport of only 2 - 58%, this represented a significant reduction compared to control eye transport (93.4 ± 3.0% intact, p = 0.001). For the BMD group, OHT-induced deficits were far less, with an average deficit of 36.9 ± 7.8% and a range of intact transport of 36 - 97%. While this range was decreased compared to control eye colliculi for the group (97.0 ± 1.5% intact, p = 0.004), BMD treatment improved transport by 124% compared to the OHT colliculi for vehicle animals (p = 0.002).

**Figure 7:**
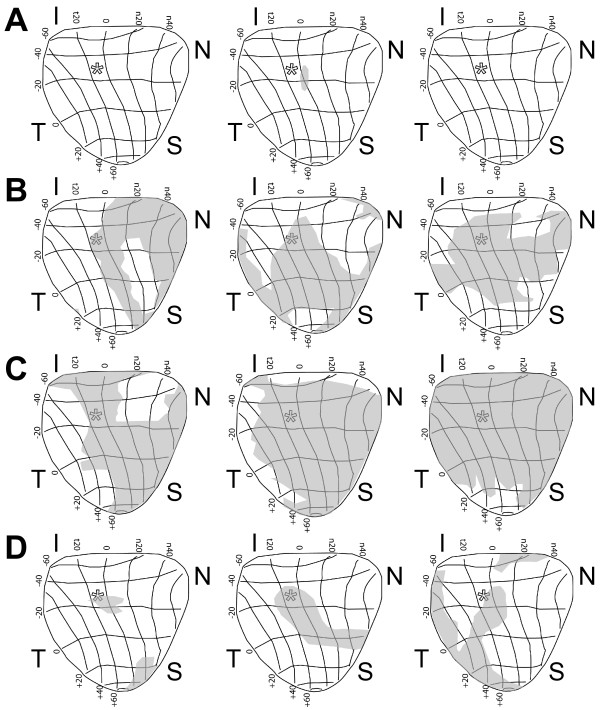
**Deficits in anterograde transport begin in the nasal retina**. Retinotopic maps of CTB transport transformed into retinal quadrant and eccentricity coordinates following Siminoff et al. (1966) [27] and Drager and Hubel (1976) [28]. (A) Colliculus maps for naïve (left), vehicle-group control (middle) and BMD-group control (right) eyes are complete (98-99%) and show location of optic disk (*). (B) Moderate transport deficits (shaded regions) in colliculi from vehicle OHT eyes with 58%, 50% and 43% intact transport (left to right respectively) appear to spread from the nasal retinal representation to the optic disk. (C) Moderate to severe transport deficits in vehicle OHT colliculi with 39%, 19% and 2% intact transport (left to right respectively) continue to spread from the optic disk to other retinal quadrants. (D) BMD treatment ameliorates most transport deficits (96% intact, left) but the same spatial pattern of progression applies for even modest deficits (84% and 80% intact, middle and right respectively). Abbreviations: I, N, S, T indicate inferior, nasal, superior and temporal quadrants of the retina, respectively.

We found earlier that the most dramatic reduction in RGC density in the retina was in the nasal quadrant (Figure 4). In other animal models of glaucoma, loss of anterograde transport to the superior colliculus is also spatially-specific, filling in one retinal sector completely before affecting another [21]. We found that typically, transport deficits due to OHT in the vehicle group were quite severe (see above), so spatial progression was difficult to assess. However, when we transformed the retinotopic maps of colliculus transport into retinal quadrant and eccentricity coordinates following Siminoff et al. [27] and Drager and Hubel [28], we found hints that deficits due to OHT begin nasally. Compared to naïve or control (Figure 7A), for vehicle colliculi, moderate OHT-induced transport deficits (42% to 57% loss) filled in from the nasal retina to the optic disk representation (Figure 7B). In vehicle colliculi with severely affected transport (>80% loss), deficits continued to spread from the nasal representation to the optic disc and into other retinal sectors. Even the more modest transport deficits from OHT eyes in the BMD-treated group appeared to follow the same geographic progression (Figure 7D).

## Discussion

In this study, we quantified how systemic delivery of BMD via subcutaneous osmotic pump protected RGCs challenged by elevated IOP in a rat model of OHT. In the absence of treatment, OHT for 8 weeks induced pathology at several levels. In the vehicle group, the worst OHT-related outcome was 70% depletion in transport of CTB to the superior colliculus (Figure 6). This deficit cannot be accounted for entirely by loss of active uptake of CTB by RGCs in the retina, which was reduced by only 44% (Figure 3). Depletion of transport was followed by a 33% loss of axons in the optic nerve (Figure 5) and finally by an average decrease in RGC somatic density of about 16% as measured by SMI31 labelling (Figure 4). In the retina, OHT was also associated with severely dystrophic axons, swollen RGC somas, and the accumulation of phosphorylated neurofilaments in dendrites (Figure 2).

These results support an OHT-induced progression in which a functional deficit precedes structural pathology. Loss of active uptake and transport of CTB was far worse than axon loss in the optic nerve, which in turn was worse than RGC somatic drop-out in the retina. This progression is similar to that described in the DBA2J mouse model of glaucoma [20,21,23]. Interestingly, RGC somatic loss as measured by SMI31 labelling was not uniform. Rather, it was worst in the nasal retina, where OHT decreased RGC density 26 to 42% across eccentricities (Figure 4). This spatial progression too is consistent with the sectorial pattern of RGC pathology observed in other glaucoma models [22,23,29,30]. Though axonal transport to the superior colliculus was severely affected by OHT, our retinotopic maps also hint of sectorial progression (Figure 7; [21]).

Although there is some earlier evidence that BMD can preserve optic nerve axons and RGC axonal transport after acute injury [9,31,32], previous studies of BMD's effect in glaucoma models have focused on retinal outcomes [11,12,16,33,34]. Following episcleral vein cautery, BMD delivered via intraperitoneal injection prevented OHT-induced loss of RGC cell bodies labelled by retrograde transport of FluoroGold from the colliculus [12,33]. In studies similar to ours in which OHT was induced by laser photocoagulation of episcleral and limbal veins for 3 weeks, BMD delivered by subcutaneous pump also improved the number of RGCs labeled retrogradely [11,16,34]. Our results are in agreement with these previous studies in that systemic BMD delivery increased RGC cell body survival (Figure 3 and Figure 4), even for our much longer period of OHT. However, given recent evidence that RGC somatic loss occurs late in disease progression [20], we examined axonal transport to the colliculus and axon survival in the nerve, both of which are challenged much earlier [21].

We found that systemic BMD treatment via subcutaneous delivery ameliorated the effects of OHT on each of our outcome measures. In the retina, BMD preserved RGC axonal and dendritic morphology (Figure 2), restored CTB uptake (Figure 3), and increased the number of SMI31+ RGCs, especially in the nasal retina where OHT-induced loss was greatest (Figure 4). In the optic nerve, BMD restored the axonal population to control levels as well (Figure 5). Finally, BMD improved anterograde axonal transport to the SC by 124% (Figure 6). As transport deficits to and from RGC targets are relevant to the progression of glaucoma [20,21], the ability of BMD to preserve optic nerve axons and axonal transport suggest its neuroprotective effects are pertinent not only at RGC soma, but throughout the entire retinal projection.

BMD is a non-subtype-selective α2-adrenergic receptor agonist. All three α2 receptors subtypes are expressed in the retina, with expression of α2A and α2B receptors by RGCs and α2B receptors by glia [35,36]. Activation of α2 receptors within the retina elicits many responses including reduced glial activation [11,37], decreased oxidative stress [38,39] and protection against apoptosis [40-42]. In addition, BMD may protect RGCs by regulating intracellular Ca^2+ ^levels and glutamate availability within the retina [16,43]. Excess glutamate can result in RGC apoptosis via overstimulation of NMDA receptors and subsequent increases in intracellular Ca^2+^. Stimulation of α2 receptors also results in the activation of the phosphatidylinositol 3 kinase (PI3K) pathway [44,45]. Activation of the PI3K pathway promotes RGC survival after various injuries [46,47], perhaps by altering gene expression [48-50], regulating protein activity [51,52], or by affecting cellular metabolism [53,54]. These and other responses make α2 agonists like BMD ideal neuroprotective agents within the retina.

A potential mechanism underling the neuroprotective effect of BMD on RGC axons involves the inactivation of glycogen synthase kinase-3 (GSK3) via the PI3K pathway. GSK3 is a constitutively active and ubiquitous kinase. In neurons, GSK3 phosphorylates microtubule-associated protein 1B, resulting in loss of stable microtubules [55]. GSK3 also phosphorylates collapsin response mediator protein-2, which promotes microtubule assembly and links tubulin heterodimers to kinesin-1 to regulate protein transport to distal regions of axons [56-58]. Also, GSK3 activation is implicated in hyper-phosphorylation of axon cytoskeletal proteins, including neurofilaments [59,60]. Interestingly, GSK3 can be inactivated by downstream kinases in the PI3K pathway [61-63], thus providing a possible link between α2 receptor activation and preservation of the axonal cytoskeleton. Activation of the PI3K pathway via treatment with BMD could counteract these changes, consequently preserving both axonal transport and structure. Since BMD applied either topically to the cornea or via systemic injection reaches the posterior segment in appreciable concentrations [64], its use could represent a viable intervention for combating early axon deficits in glaucoma.

## Conclusions

Ocular hypertension in rats resulted in a substantial decline in RGC axonal transport to the superior colliculus, diminished axon survival in the optic nerve, and reduced RGC density in the retina, especially in the nasal quadrant. Systemic treatment with BMD significantly improved axonal transport and survival and either prevented or decreased loss of RGC density across retinal quadrants. As transport deficits to and from RGC targets are relevant to the progression of glaucoma, the ability of BMD to preserve optic nerve axons and axonal transport suggest its neuroprotective effects are pertinent not only at the cell body, but throughout the entire retinal projection as well.

## Methods

### IOP elevation and brimonidine delivery

Unilateral IOP elevation in male Sprague-Dawley rats (weight range, 350 - 400 g) was achieved by laser photocoagulation of episcleral and limbal veins as described previously [11]. Rats were anesthetized with a mixture of ketamine (50 mg/kg), acepromazine (1 mg/kg), and xylazine (25 mg/kg) and two laser treatments were performed 1 week apart in order to achieve persistent IOP elevation. IOP was measured with a tonometer (TonoLab; Colonial Medical Supply, Franconia, NH). BMD (1 mg/kg/day) or vehicle phosphate-buffered saline (PBS) was administered systemically and continuously using an osmotic pump (Alzet; Durect, Cupertino, CA) inserted subcutaneously on the back of 16 animals per group. This concentration and mode of delivery was based on earlier results comparing 0.5 mg/kg/day vs. 1 mg/kg/day for efficacy in protecting RGC cell bodies during OHT [11]. Treatment began at the time of initial IOP elevation and continued for 8 weeks. Age-matched naïve control rats (n = 16) were also included for comparison.

### **Cholera toxin **β **injection and tissue preparation**

Forty-eight hours prior to sacrifice, rats were anesthetized with a mixture of ketamine (50 mg/kg) and xylazine (25 mg/kg) and eyes anesthetized locally using topical application of oxybuprocaine chlorhydrate [65]. The hypertensive eye received an intravitreal injection (6 μl) of 0.5 mg Cholera toxin subunit β (CTB) conjugated to Alexa Fluor 594 (Molecular Probes, CA) while the control eye received a similar injection of CTB conjugated to Alexa Fluor 488 (Molecular Probes, CA) following earlier studies [21]. After the 48 hour period, animals were deeply anesthetized with a lethal intraperitoneal injection of sodium pentobarbital (150 mg/kg) and perfused intracardially with warm (37°C) heparinized saline followed by 300 ml of Zamboni's fixative at 4°C. Brains were cryoprotected overnight in 30% sucrose/PBS and 50 μm coronal sections were taken through the midbrain and mounted on gelatin-coated slides. Retinas were dissected from the eye and vitreous removed after treatment with collagenase (400 units/ml) at 37°C for 15 minutes. Sections of optic nerve 2-3 mm proximal to the globe were isolated, post-fixed and prepared for embedding and semi-thin sectioning as previously described [66,67].

### Retinal ganglion cell number and CTB uptake

RGCs were immuno-labelled using antibodies against phosphorylated neurofilament-heavy (SMI31, Sternberger Monoclonal Incorporated, Baltimore, MD) following our published protocol [21]. Confocal images (0.101 mm^2^) were captured on an Olympus FV-1000 inverted confocal microscope along the midline of each retinal quadrant at 1 to 4 mm from the optic disc. The number of SMI31-positive and CTB-positive RGCs per image were counted and RGC density calculated as cells per mm^2^.

### Axon quantification

A 2- to 3-mm section of optic nerve proximal to the globe was isolated, post-fixed for 1 hour in 4% paraformaldehyde, and prepared for embedding and sem-ithin sectioning [66]. Sections were stained with para-phenylenediamine (PPD) and then photographed using 100× oil-immersion and differential interference contrast optics. Photomicrographs of each optic nerve section were collected as a montage using an Olympus Provis AX70 microscope with motorized X-Y-Z stage and a digital CCD video camera. Each montage was contrast and edge-enhanced using the ImagePro software package (Media Cybernetics, CA). An additional routine was used to identify and count each axon in the montage for which a myelin sheath could be identified. We used this information to calculate the mean local axon density for each section of nerve. This was multiplied by the cross-sectional area of the nerve section to obtain an estimate of the total number of axons as described [66].

### Measurement of anterograde transport

Alternate sections of the brain containing superior colliculus were photographed using a Spot-RT camera on an Olympus AX-70 upright microscope and intensity of label was quantified using ImagePro (Media Cybernetics, Bethesda, MD) as described previously [21]. After normalizing with respect to background, intensity was recorded based on mediolateral location in the section. Intensity calculations from alternate sections were then combined to form a colorimetric representation of CTB signal across the retinotopic collicular map. For each colliculus, we determined the fraction of intact retinotopic map, defined as the percent area with CTB signal ≥70% maximum.

### Statistical analysis

Unless otherwise indicated, all data are presented as the mean ± the standard error of the mean (SEM). SigmaPlot for Windows version 11.0 (Systat Software, Inc,; Chicago, IL) was used to calculate p values in comparing data using either ANOVA or t tests for data meeting criteria for normalcy or using non-parametric rank statistics for data failing normalcy.

## List of abbreviations

BMD: brimonidine; CTB: cholera toxin subunit β; cAMP: cyclic adenosine monophosphate; GSK3: glycogen synthase kinase-3; IOP; intraocular pressure; NMDA: N-methyl-D-aspartic acid; OHT: ocular hypertension; PI3K: phosphatidylinositol 3 kinase; RGCs: retinal ganglion cells.

## Competing interests

WSL, SDC and DJC received funding from Allergan Inc. (Discovery Research Grant to DJC) for these studies. LR and LAW were employed by Allergan Inc. during these studies. All experimental procedures were approved by the Vanderbilt University Medical Center Institutional Animal Care and Use Committee.

## Authors' contributions

WSL carried out the retinal immunocytochemistry, cell counts, statistical analysis, and helped to draft the manuscript. LR carried out the IOP elevation, BMD delivery, CTB intravitreal injections and tissue acquisition. SDC carried out the anterograde transport measurements, statistical analysis and helped to draft the manuscript.

LAW conceived of the study, and participated in its design and coordination.

DJC participated in the study design, coordination and analysis and oversaw the preparation of the manuscript. All authors read and approved the final manuscript.
